# Comparative Study of Molecular Descriptors and AI-Based Embeddings for Toxicity Prediction

**DOI:** 10.1021/acs.chemrestox.5c00289

**Published:** 2025-11-14

**Authors:** Magnus Gray, Leihong Wu

**Affiliations:** Division of Bioinformatics and Biostatistics, National Center for Toxicological Research, 4136 U.S. FDA, 3900 NCTR Rd, Jefferson, Arkansas 72079, United States

## Abstract

Accurate toxicity prediction is a critical component of pharmaceutical development and regulatory safety evaluation, traditionally relying on molecular descriptor-based models. This study compares the performance of descriptor-based features (Mordred, RDKit) with embeddings from ten AI language models applied to SMILES strings, chemical names, and simple descriptions, using logistic regression classifiers across the Tox21, ClinTox, and DILIst datasets. For the Tox21 dataset, Mordred achieved the highest average ROC-AUC of 0.855, outperforming language models. However, on specific endpoints, language models showed competitive performance, with MolBERT reaching an average ROC-AUC of 0.801 for SMILES-based embeddings. In contrast, language models outperformed descriptor models on the ClinTox dataset. While RDKit achieved an ROC-AUC of 0.721, GPT-3 reached 0.996 by using simple descriptions. Similarly, for the DILIst dataset, language models surpassed descriptor models, with GPT-3 achieving an ROC-AUC of 0.806 using chemical names, compared to RDKit’s 0.620. These results demonstrate the promise of AI language models in predictive toxicology, particularly for specific toxicity endpoints and datasets. While molecular descriptors remain robust for multiendpoint predictions like Tox21, language models show superior performance on focused toxicity classifications such as ClinTox and DILIst. This study supports the future integration of molecular descriptors with textual embeddings to enhance overall performance and adaptability across diverse toxicity prediction tasks.

## Graphical abstract

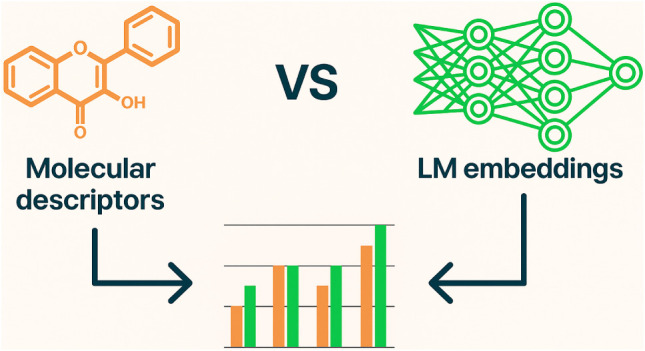

## Introduction

Ensuring public health and safety requires accurate documentation and research on drug and chemical characteristics, particularly toxicity. Regulatory bodies like the U.S. Food and Drug Administration mandate drug labeling documents that provide information on indications, adverse reactions, and warnings and precautions.^1^ Additionally, molecular representations—such as SMILES (Simplified Molecular Input Line Entry System) strings^2^—play an important role in understanding a compound’s chemical structure, biological interactions, and toxicity potential. These structured data sources enable the development of predictive models for toxicity assessment.

To evaluate toxicity prediction models, well-established datasets such as Tox21 have been instrumental. The Tox21 dataset consists of over 7,600 chemical compounds labeled for 68 toxicity targets, including key endpoints such as the neurotoxicity target “ache-p3” and the developmental toxicity target “shh-3t3-gli3-antagonist-p1”.^3^ Similarly, the ClinTox dataset provides toxicity classifications (i.e., “toxic” or “non-toxic”) for over 1,400 compounds,^4^ while the DILIst dataset provides toxicity classifications for over 1,200 compounds based on their potential for causing drug-induced liver injury (DILI).^5^ These datasets allow researchers to assess and compare predictive models, guiding the development of optimal toxicity prediction frameworks.

Traditionally, molecular descriptor-based models—such as those leveraging Mordred^6^ or RDKit^7^—have set the standard for toxicity prediction. These models extract thousands of molecular features, including molecular weight, atom count, and aromaticity, providing a comprehensive chemical profile that often results in high predictive performance. However, with the rise of deep learning and natural language processing (NLP), artificial intelligence (AI)-powered language models offer an alternative approach by generating text-based molecular embeddings. These embeddings, derived from representations like SMILES strings, chemical names, and simple descriptions (e.g., ChEBI^8^), capture contextual relationships between molecules. Recent work has highlighted the emergence of large language models (LLMs) in chemistry, demonstrating their ability to capture structural and functional information relevant to molecular property prediction. For example, Text2Mol learns a joint embedding space between molecules and natural language descriptions, enabling cross-modal retrieval of compounds from free-text queries.^9^ Similarly, general-purpose LLMs such as GPT-3 ^10^ and Llama^11^ derivatives, when applied to chemical names and descriptions, have shown promise in encoding biomedical and toxicological knowledge that extends beyond purely structural inputs. These approaches complement earlier chemically specialized transformers (e.g., ChemBERTa,^12^ MolBERT,^13^ MoLFormer^14^) but differ in scale and scope by leveraging billions of parameters and diverse textual corpora. Together, these LLM-based studies point to a growing frontier where cross-modal and large-scale pretrained models provide new baselines and motivate comparative evaluations in molecular property and toxicity prediction.

In this study, we systematically compare molecular descriptor-based models with text-based embeddings generated from ten language models, including BERT,^15^ ChemBERTa (v1),^12^ GPT-3,^10^ and Llama-3,^11^ to predict toxicity using the Tox21, ClinTox, and DILIst datasets. While our results indicate that molecular descriptor-based models generally outperform language model embeddings, our findings also suggest that language models may capture toxicity-related patterns missed by descriptor-based approaches. In some endpoints, where descriptor models perform poorly, language embeddings may provide competitive predictive power, highlighting their potential utility in toxicity assessment. By comparing computational toxicology with NLP-driven embeddings, this study seeks to determine whether language models can complement or surpass traditional descriptor-based approaches in toxicity prediction.

## Methods

To evaluate the performance of toxicity prediction models, this study utilized the Tox21, ClinTox, and DILIst datasets.^3–5^ First, Tox21 includes over 7,600 compounds labeled for 68 toxicity targets. These endpoints correspond to various biological targets relevant to chemical safety assessments. Each compound is classified as active or inactive for each of the endpoints, with the active category further broken down into “active agonist” and “active antagonist.” To enable a binary classification approach, the positive class was defined based on which active subcategory was more common, with the negative class being defined as the inactive label. To ensure minimally stable per-endpoint evaluation and to avoid endpoints with very few positives or negatives, we retained only Tox21 endpoints that originally contained at least 20 positive and 20 negative examples; this filtering reduced the dataset from 68 to 29 endpoints. For each retained endpoint, we then constructed a balanced per-endpoint dataset by randomly sampling exactly 20 positives and 20 negatives when more examples were available (i.e., 40 molecules per endpoint); endpoints with fewer than 20 examples per class were excluded. The resulting per-endpoint sets were split into training and test partitions following the published train/test splits of Wu et al.,^16^ originally created with a random sampling approach.

Second, the ClinTox dataset classifies the toxicity of over 1,400 drugs and chemicals as either “toxic” or “non-toxic.” Following the extraction of the appropriate molecular representations for each compound, this dataset was reduced to 655 samples, with 591 in the training split and 64 in the testing split, adhering to the published 80/10 train/test splits (omitting the 10% validation split) of Wu et al.,^4^ which were created with a scaffold split approach. Third, the DILIst dataset is a benchmark for DILI toxicity that consists of over 1,200 drugs labeled as either “DILI Positive” or “DILI Negative.” After extracting the molecular representations for each compound and processing the data, this dataset was reduced to 1022 compounds, which were split 80% for training and 20% for testing using Bemis–Murcko scaffolds.

For each dataset, the applied molecular representations include canonical SMILES strings and preferred chemical names. Additionally, simple descriptions from the ChEBI^8^ database were retrieved using the PubChem^17^ API, allowing for a more comprehensive comparison of text-based molecular representations alongside structural data. Table 1 provides more details about these textual data sources as well as examples of each. Altogether, Tox21, ClinTox, and DILIst serve as the benchmarks for assessing and comparing modeling approaches for the task of toxicity prediction.

**Table 1:** Explanations and Examples of Data Sources for the Language Model Embeddings

Data Source	Description	Notable Aspects	Examples
**Canonical SMILES Strings**	Simplified molecular input line entry system (SMILES) encodings structured as text strings.	•Directly encode molecular structure;	•[O−][N+](=O)C1=CC=C(Cl)C=C1
		•Sensitive to syntax variations	•NC1=CC=C(C=C1)[N+]([O−])=O
**Chemical Names**	Common names used to reference chemical compounds.	•Human-readable;	
		•Variability in naming conventions;	•1-Chloro-4-nitrobenzene
		•May not encode full structural details	•4-Nitroaniline
**Simple Descriptions**	Sentence-based descriptions of chemicals from the ChEBI database.	•Contains biological or functional information;	•4-Chloronitrobenzene is a C-nitro compound.
		•May introduce information unrelated to toxicity	•4-nitroaniline is a nitroaniline carrying a nitro group at position 4. It has a role as a bacterial xenobiotic metabolite.

Two modeling approaches were examined: molecular descriptor-based models and language model-generated embeddings. Mordred^6^ and RDKit^7^ were used to compute molecular descriptors that capture structural and physiochemical properties, which were then used to train logistic regression and random forest classifiers as a baseline. The second approach involved generating embeddings from BERT (“bert-base-uncased”^18^), ChemBERTa (“ChemBERTa-zinc-base-v1”^19^), PharmBERT (“PharmBERT-uncased”^20^), RxBERT (“rxbert-v1”^21^), MolBERT (“Molbert_100epochs”^22^), MoLFormer (“MoLFormer-XL-both-10pct”^23^), Llama-3 (“Llama-3.1–8B”^24^, “Llama-3.2–1B”^25^, and “Llama-3.3–70B-Instruct”^26^), and GPT-3 (“text-embedding-3-large”^27^). All listed language models were used as pretrained (fixed) embedding providers in this study: we did not fine-tune model weights on the toxicity tasks. ChemBERTa, MolBERT, MoLFormer, RxBERT, and PharmBERT were specifically pretrained with chemically focused corpora, while the general-purpose language models (e.g., BERT, Llama, and GPT-3) were pretrained without this focus.

The selected language models processed the SMILES strings, chemical names, and simple descriptions, producing vector embeddings (using the mean pooling method) that served as input features for separate logistic regression and random forest models. For clarification, the language models developed to specifically process SMILES strings (i.e., ChemBERTa, MolBERT, and MoLFormer) were not utilized for the other two textual data sources. The effectiveness of the models was measured using the Receiver Operating Characteristic – Area Under the Curve (ROC-AUC) score, which assessed the classification performance. An additional comparative analysis was conducted for the Tox21 endpoints using a Mean Absolute Z-score (MAZ) metric, which quantifies how closely each language model’s endpoint-level ROC-AUCs align with descriptor-based models (Mordred and RDKit). For each endpoint *i*, let *X*_LM,*i*_ be the LM’s ROC-AUC, and let μ_D_ and σ_D_ be the mean and standard deviation of a descriptor model’s ROC-AUCs across endpoints. The per-endpoint Z-score is *Z*_*i*_ = (*X*_LM,*i*_ – μ_D_)/σ_D_. The MAZ is then the mean of |*Z*_*i*_| across endpoints, so lower MAZ indicates closer alignment of an LM’s endpoint-level behavior with the descriptor baseline. For each LM, we report MAZ versus Mordred and versus RDKit and summarize the findings in the main results (with the detailed table available in Table S1

The study design is illustrated in Figure 1. This study aimed to determine whether language model embeddings could match or surpass molecular descriptors in predictive performance and to identify cases in which text-based representations provided additional insights. Moreover, this study primarily focuses on toxicity classification tasks; regression tasks (continuous toxicity endpoints) were not included but are an important complementary evaluation that should be addressed in future work.

**Figure 1:**
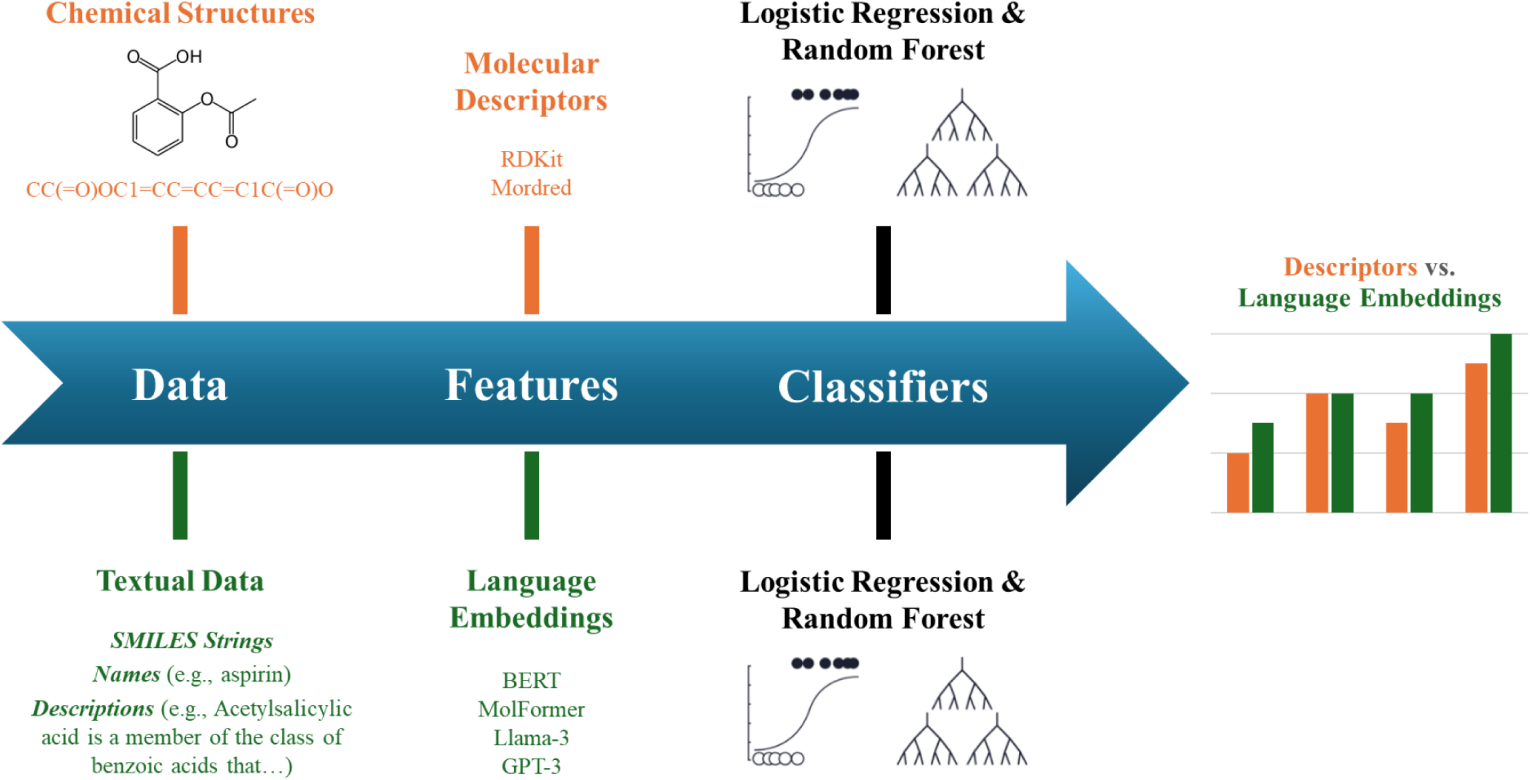
Study design. For each dataset, we generated (A) molecular descriptors (Mordred, RDKit) and (B) embeddings from multiple language models for three textual data sources (SMILES, names, descriptions). Features were pooled and provided as inputs to separate logistic regression and random forest classifiers. Performance was evaluated per endpoint (Tox21) or per dataset (ClinTox, DILIst) using ROC-AUC.

All analyses were conducted in Python (v. 3.8.20). Data processing was performed using the pandas (version 2.0.3) library. Molecular descriptors were generated using the Mordred (v. 1.2.0) and RDKit (v. 2024.3.5) libraries, while language model embeddings were obtained using the Hugging Face Transformers (v. 4.46.3) library for local models and the OpenAI API (v. 1.104.2) for GPT-3 (text-embedding-3-large). The logistic regression and random forest models were implemented with scikit-learn (v. 1.3.2). For these classifiers, default hyperparameters were utilized, except for setting the maximum iterations (max_iter) to 1000 for logistic regression and the number of trees (n_estimators) to 100 for random forest, following standard practice to ensure reliable training. Experiments involving local language models were run on a high-performance computing (HPC) server equipped with 8× NVIDIA Tesla V100-SXM2-32GB GPUs. GPT-3 embeddings were retrieved via API requests, with typical rate limits (∼60 requests/min) and latency expected for remote inference. While detailed runtime or cost data were not recorded, the resources used reflect realistic conditions for academic or applied machine learning research. All processed datasets and scripts for model evaluation are available at https://github.com/magnus-gray/Embedding4Tox.

## Results

The performance of molecular descriptor-based models and language model embeddings was evaluated using both logistic regression and random forest classifiers trained on features derived from each approach. ROC-AUC scores were used as the primary metric to assess classification performance on the Tox21, ClinTox, and DILIst datasets.

### Tox21 Results

First, for the Tox21 dataset, Mordred achieved the highest predictive performance, with an average (across all endpoints) ROC-AUC of 0.855 for the logistic regression classifier, reaffirming the effectiveness of molecular descriptors for toxicity prediction. RDKit also demonstrated strong performance, though slightly lower than that of Mordred, with an average ROC-AUC of 0.839 for this classifier. Among the language models, the results varied depending on the molecular representations used. MolBERT exhibited the highest performance for SMILES-based embeddings, achieving an average ROC-AUC of 0.801 for the logistic regression classifier. GPT-3 performed the best when using chemical names, reaching 0.738, while Llama-3.1-8B produced the strongest results for simple descriptions at 0.734.

Using a random forest classifier as a sensitivity check, descriptor features improved over the logistic regression baselines (e.g., Mordred: 0.855 → 0.883; RDKit: 0.839 → 0.863 on Tox21), consistent with nonlinear interactions among physicochemical descriptors. In contrast, LM embeddings changed little and inconsistently with random forest (e.g., MolBERT 0.801 → 0.795 on Tox21), suggesting much of their nonlinearity is already encoded in the embeddings and captured adequately by a linear head. A cross-dataset summary of the random forest results appears in Figure S1.

### Specific Endpoint Results

Although language model embeddings did not outperform descriptor-based models overall for the Tox21 dataset, this benchmark contains 29 endpoints after processing, and some of these endpoints showed cases where they provided comparable or superior performance. Figure 2 illustrates the performance—as the ROC-AUC scores of the logistic regression classifier—of the descriptor models and the top-performing language models for each textual data type (e.g., SMILES, chemical names, and simple descriptions) across each Tox21 endpoint. For clarification, this figure and the ones that follow, the label “Top LM for [‘SMILES’, ‘Names’, or ‘Descriptions’]” refers to the language model that, on average, achieved the highest ROC-AUC scores for the specific dataset with the respective textual type.

**Figure 2:**
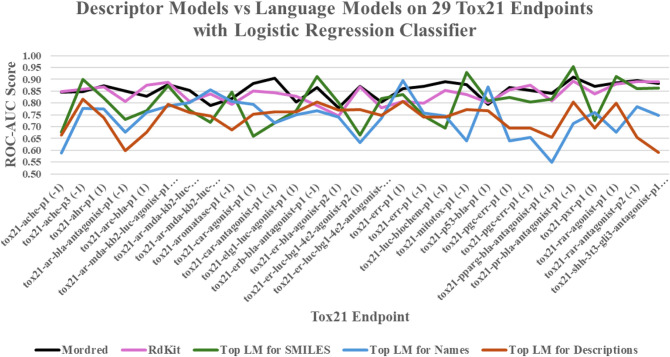
Line chart of ROC-AUC scores on 29 Tox21 endpoints, for the logistic regression classifier. Top LM for SMILES = MolBERT, Top LM for Names = GPT-3, Top LM for Descriptions = Llama-3.1-8B.

To assess statistical differences in model performance across the 29 Tox21 endpoints, we opted for a paired nonparametric approach because the endpoints are often correlated and the distribution of per-endpoint ROC-AUC differences may violate normality assumptions. Specifically, we performed two-sided Wilcoxon signed-rank tests (paired across endpoints), reported the median of the paired ROC-AUC differences (baseline – comparator) with 95% bootstrap confidence intervals (5,000 resamples) to quantify effect size and uncertainty, and applied Benjamini–Hochberg correction to control the false discovery rate. Results are summarized in Figure 3 as a forest plot showing median ROC-AUC differences of ±95% bootstrap CI with BH-adjusted *p*-values annotated. Using Mordred as the baseline, the median ROC-AUC differences (Mordred – other) across the 29 endpoints are RDKit = 0.0182 (95% CI 0.0008–0.0332; BH *p* = 0.0224), Top LM (SMILES) = 0.0361 (95% CI 0.0043–0.0711; BH *p* = 0.0068), Top LM (Names) = 0.1100 (95% CI 0.0721–0.1731; BH *p* < 1 × 10^–5^), and Top LM (Descriptions) = 0.1302 (95% CI 0.0840–0.1498; BH *p* < 1 × 10^–7^). These results support the conclusion that descriptor-based features outperform the evaluated language-model embeddings on average across Tox21 while providing more robust statistical inference.

**Figure 3:**
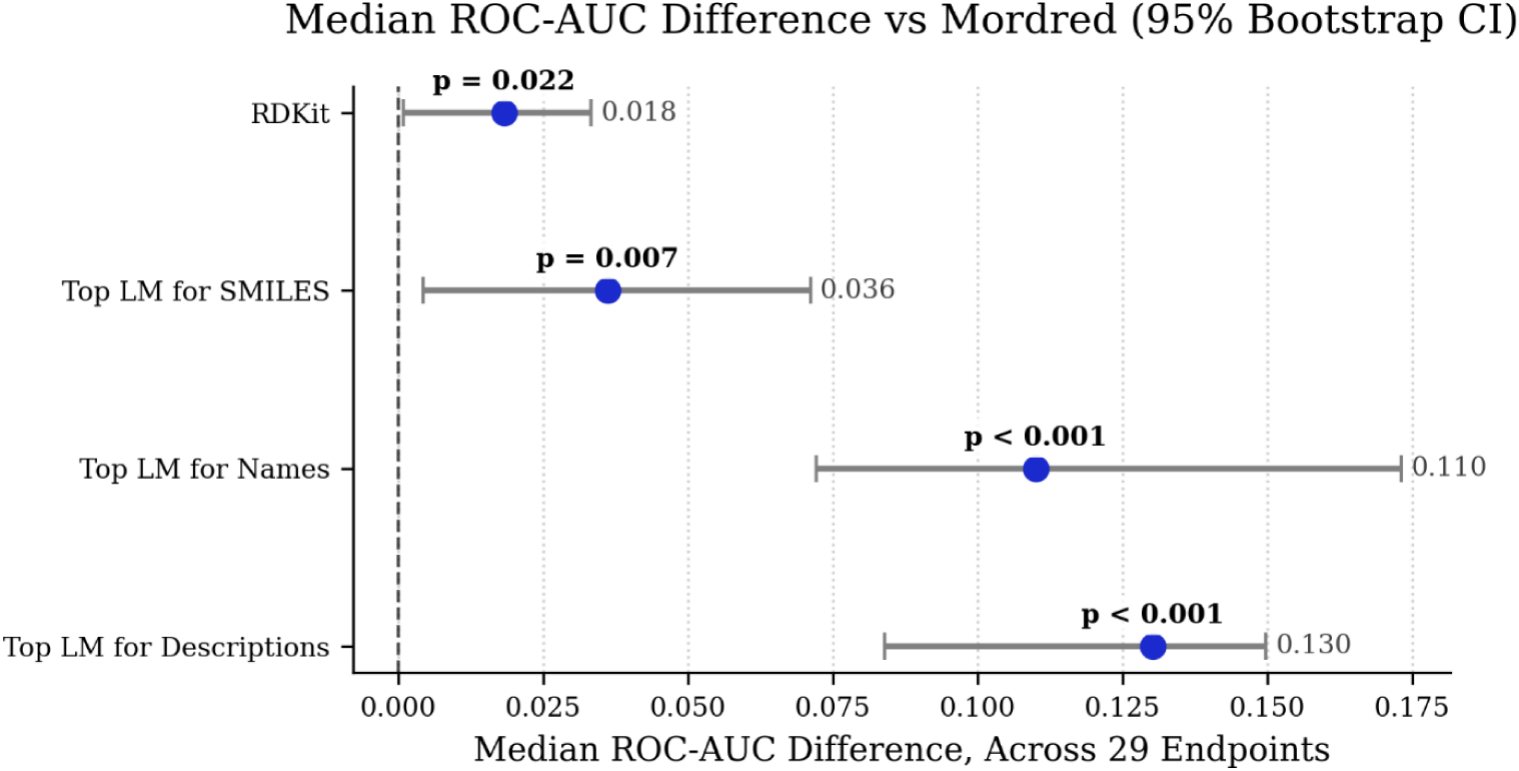
Forest plot of median ROC-AUC differences between models, using Mordred as a baseline and 95% bootstrap confidence interval. Top LM for SMILES = MolBERT, Top LM for Names = GPT-3, Top LM for Descriptions = Llama-3.1-8B.

Table 2 summarizes several instances where language models outperformed descriptor models, with ROC-AUC scores for the top-performing descriptor and language models, as well as the average ROC-AUC for all of the evaluated language models. These results suggest that even within a dataset where traditional descriptors dominate, there are nuanced biological targets or mechanisms where NLP-derived representations may excel. In such cases, the flexibility of language models to incorporate indirect associations and latent patterns becomes advantageous.

**Table 2:** Tox21 Endpoints Where Language Models Outperform Descriptor Models for Logistic Regression Classifier

Tox21 Endpoint	Language Embedding Type	Top Descriptor Model	Top Language Model	Average Language Model
tox21-aromatase-p1 (−1)	SMILES	0.819 (Mordred)	0.898 (MoLFormer)	0.844
tox21-er-luc-bg1–4e2-antagonist-p1 (−1)	SMILES	0.805 (Mordred)	0.898 (ChemBERTa)	0.805
tox21-rar-agonist-p1 (1)	SMILES	0.884 (Mordred)	0.945 (GPT-3)	0.893
tox21-ar-mda-kb2-luc-agonist-p1 (1)	SMILES	0.888 (RDKit)	0.922 (BERT)	0.890
tox21-p53-bla-p1 (1)	SMILES	0.802 (RDKit)	0.810 (MolBERT)	0.713
	Names	0.802 (RDKit)	0.867 (GPT-3)	0.667
	Descriptions	0.802 (RDKit)	0.875 (GPT-3)	0.713

To further illustrate and compare the descriptor-based models and language models on the Tox21 dataset, Figure 4 presents a scatter plot comparing the Tox21 ROC-AUC scores for the closest-performing language model for each textual data type against Mordred (the best-performing descriptor-based model). The plot highlights a strong correlation between MolBERT (for the SMILES-based representations) and Mordred, suggesting that this language model captures some of the structured chemical properties traditionally modeled through molecular descriptors. Conversely, models with lower average ROC-AUC scores, such as the chemical name-based representations from GPT-3, exhibit weaker alignment, reinforcing the variability in language model embeddings across different Tox21 endpoints. This variability further supports the idea that not all text-based representations carry equal weight in toxicity prediction tasks. SMILES, being a direct encoding of molecular structure, aligns more naturally with descriptors, whereas chemical names and descriptions may introduce ambiguity or abstraction that reduces signal fidelity. Nonetheless, in contexts where structural data are incomplete or absent—such as legacy drug databases or clinical notes—these alternative embeddings may still offer valuable predictive utility.

**Figure 4:**
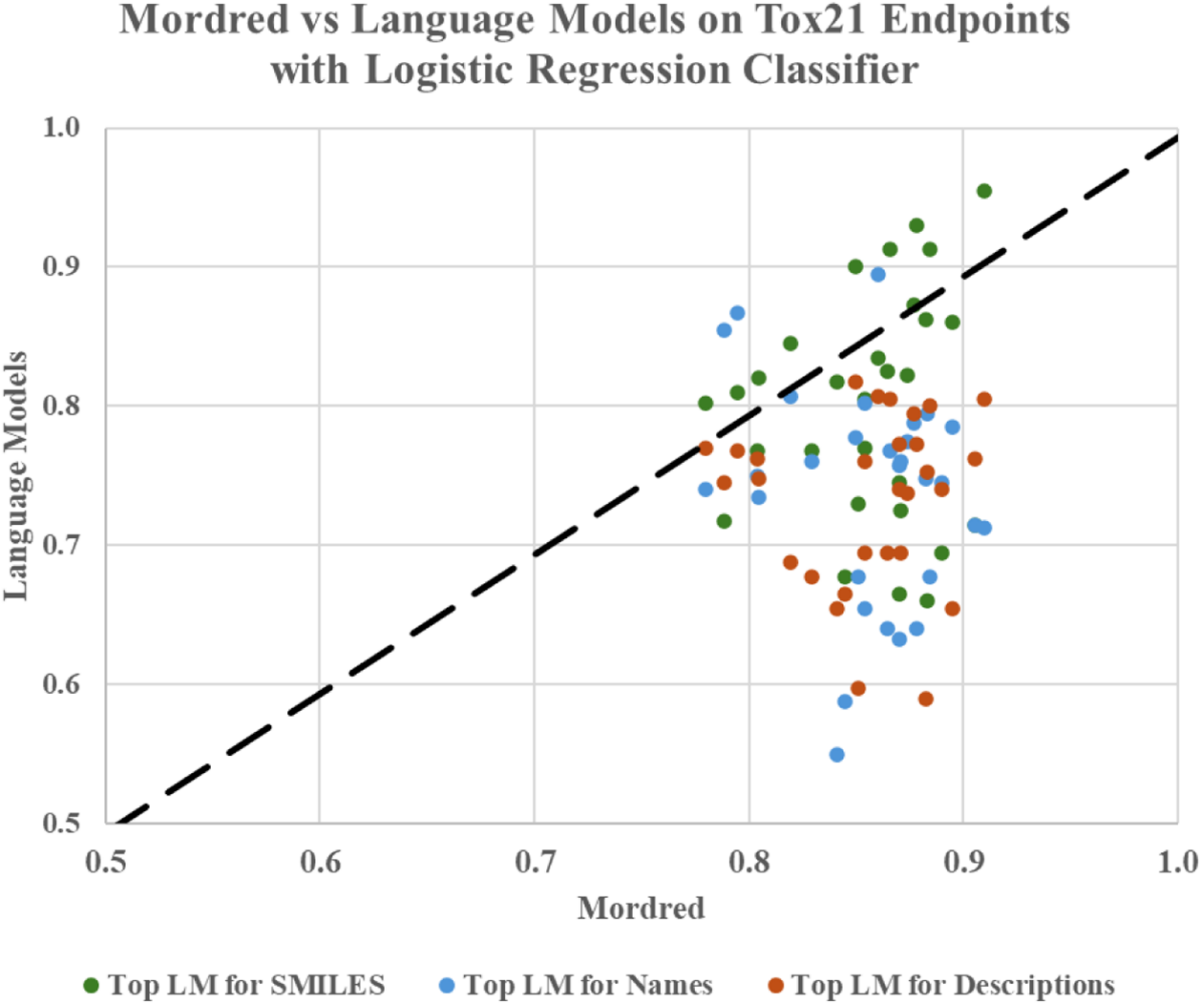
Scatter plot of relationship between 29 Tox21 endpoint ROC-AUC scores of Mordred and closest-performing language models, for the logistic regression classifier. Top LM for SMILES = MolBERT, Top LM for Names = GPT-3, Top LM for Descriptions = Llama-3.1-8B.

A complementary MAZ analysis indicates that SMILES-trained MolBERT has the lowest MAZ versus Mordred/RDKit, i.e., its endpoint-level ROC-AUCs most closely track descriptor patterns; for chemical names and simple descriptions, GPT-3 and Llama-3.1-8B show the best (lower) MAZ values, respectively. This supports the findings shown in Figure 4: SMILES embeddings align most strongly with descriptor behavior, while name/description embeddings diverge more. Full MAZ results are available in Table S1.

These results suggest that while current molecular language models have yet to match descriptor-based approaches in overall performance on Tox21, they still capture useful chemical information—particularly from structured representations like SMILES. The superior performance of MolBERT on SMILES further supports the idea that pretraining on chemically rich text allows these models to learn context-aware features that can generalize across toxicity endpoints. Despite these promising results, none of the model embeddings outperformed molecular descriptor-based models overall. However, for certain toxicity endpoints, some language model embeddings provided comparable or superior performance. This indicates that while descriptor models may remain the gold standard in broad toxicity prediction, language models are capable of capturing nuanced domain knowledge that may be particularly useful in specialized cases.

### ClinTox and DILIst Results

Second, for the ClinTox dataset, the language models showed more promising results, with the top-performing language models each outperforming the descriptor models of Mordred and RDKit, which obtained ROC-AUC scores of 0.604 and 0.721, respectively. In more detail, the top-performing language models for the SMILES strings, chemical names, and simple descriptions—BERT, Llama-3.1-8B, and GPT-3—obtained ROC-AUC scores of 0.900, 0.883, and 0.996, respectively. Under random forest, ClinTox results for LMs were similarly strong (e.g., GPT-3 0.996 → 0.999), while descriptors showed smaller gains than on Tox21, preserving the LM advantage on this dataset (see Figure S1). These high values, particularly the near-perfect score from GPT-3, highlight the potential of large language models to synthesize and leverage semantic and structural cues present in diverse textual representations. The results imply that ClinTox, which is centered on clinical toxicity, may benefit more from the type of implicit contextual knowledge that language models can extract from natural language sources, such as drug labels or adverse event reports.

A similar trend was observed for the third dataset, DILIst. For this dataset, Mordred and RDKit produced ROC-AUC scores of 0.591 and 0.580, respectively. Yet, the top-performing language models for the three text data types produced ROC-AUC scores of 0.622 (Llama-3.2–1B), 0.830 (GPT-3), and 0.701 (Llama-3.1–8B). As on Tox21, random forests yielded modest improvements for descriptor features on DILIst and mixed/noisy changes for LM embeddings (see Figure S1), leaving the main conclusion the same. Compared with descriptor-based approaches, language models showed a clear advantage for DILIst, likely due to their capacity to model the subtle linguistic patterns associated with drug-induced liver injury. This suggests that language-derived features might be particularly suited to capturing the mechanistic and clinical nuances of certain toxicity types that may not be well-reflected in physicochemical descriptors.

### Ablation Analysis: Descriptor-Embedding Fusion

Ablation experiments were conducted on the DILIst dataset that probe whether simple feature fusion—concatenating RDKit descriptors to language-model embeddings—changes predictive performance. In more detail, fusion features were formed by horizontally concatenating the RDKit descriptor matrix to each language-model embedding matrix (reported as “SMILES + RDKit”, “Names + RDKit”, and “Descriptions + RDKit”). Logistic regression classifiers were trained and evaluated over five seeds using the same pipelines as previously described. Results are reported as Mean (SD) in Table S2. In addition, individual compounds—where both descriptor sets failed but language-model embeddings succeeded—were analyzed; a small set of representative cases is listed in Table S3.

Across runs, Table S2 shows that language-model embeddings generally achieved a higher mean ROC-AUC on DILIst than descriptor-only features (RDKit, Mordred), so the principal conclusion holds under these ablation conditions. Concatenating RDKit descriptors with language-model embeddings produced variable results. For smaller LMs like BERT and ChemBERTa, the concatenation of RDKit descriptors typically led to slight increases of performance but very rarely led to performance greater than RDKit by itself. For larger models like Llama-3.1-8B, this concatenation typically had negligible effects, indicating that LLMs are less typical predictive models and more knowledge synthesis tools. Nonetheless, the simple concatenation of descriptor features does not yield promising results, and thus, different approaches should be considered in future research.

Finally, the compound-level case studies (Table S3) illustrate typical situations where textual cues available in names/descriptions appear to enable correct LM predictions, while descriptor vectors alone do not—for example, lexical tokens denoting known drug classes or functional groups that LMs have associated with toxicity during pretraining. Overall, this ablation corroborates the main findings of the primary DILIst experiment, in addition to two other key findings: (a) simple descriptor and LM embedding fusion can yield slight improvements; and (b) LMs often leverage lexical/class-level signals in names or descriptions that are not directly captured by numeric descriptors. These observations motivate future work on principled hybrid models and targeted fine-tuning to better combine the complementary strengths of structured descriptors and text-derived embeddings.

### Cross-Benchmark Summary and Key Findings

Altogether, these results show that language models can surpass traditional descriptor-based models in specific toxicity prediction tasks, demonstrating that AI has the potential to lead the future of toxicology. It also emphasizes the importance of selecting the right data representation for the task at hand, as the optimal model may depend heavily on the type of information that needs to be extracted.

To illustrate these findings, Figure 5 presents a comparison of average ROC-AUC scores for the Tox21, ClinTox, and DILIst datasets across all models for the logistic regression classifier (see Figure S1 for the random forest equivalent), with error bars for the Tox21 results based on the standard deviation of the individual endpoint results. Only the top-performing language models for each textual data type (e.g., SMILES, chemical names, and simple descriptions) are shown in the bar chart. This chart highlights the overall strength of molecular descriptor-based models while also demonstrating the competitive performance of language models, especially for the ClinTox and DILIst datasets. It is worth noting that language models exhibit increased performance variance across datasets and input types, suggesting that their generalization ability may be more dataset-dependent compared to traditional descriptors. Furthermore, the consistently strong performance of GPT-3 on natural language inputs reinforces the utility of transfer learning in toxicology, particularly when using models trained on extensive biomedical corpora. The language models’ strong results suggest that information captured through text-based representations can provide valuable insights for toxicity prediction.

**Figure 5:**
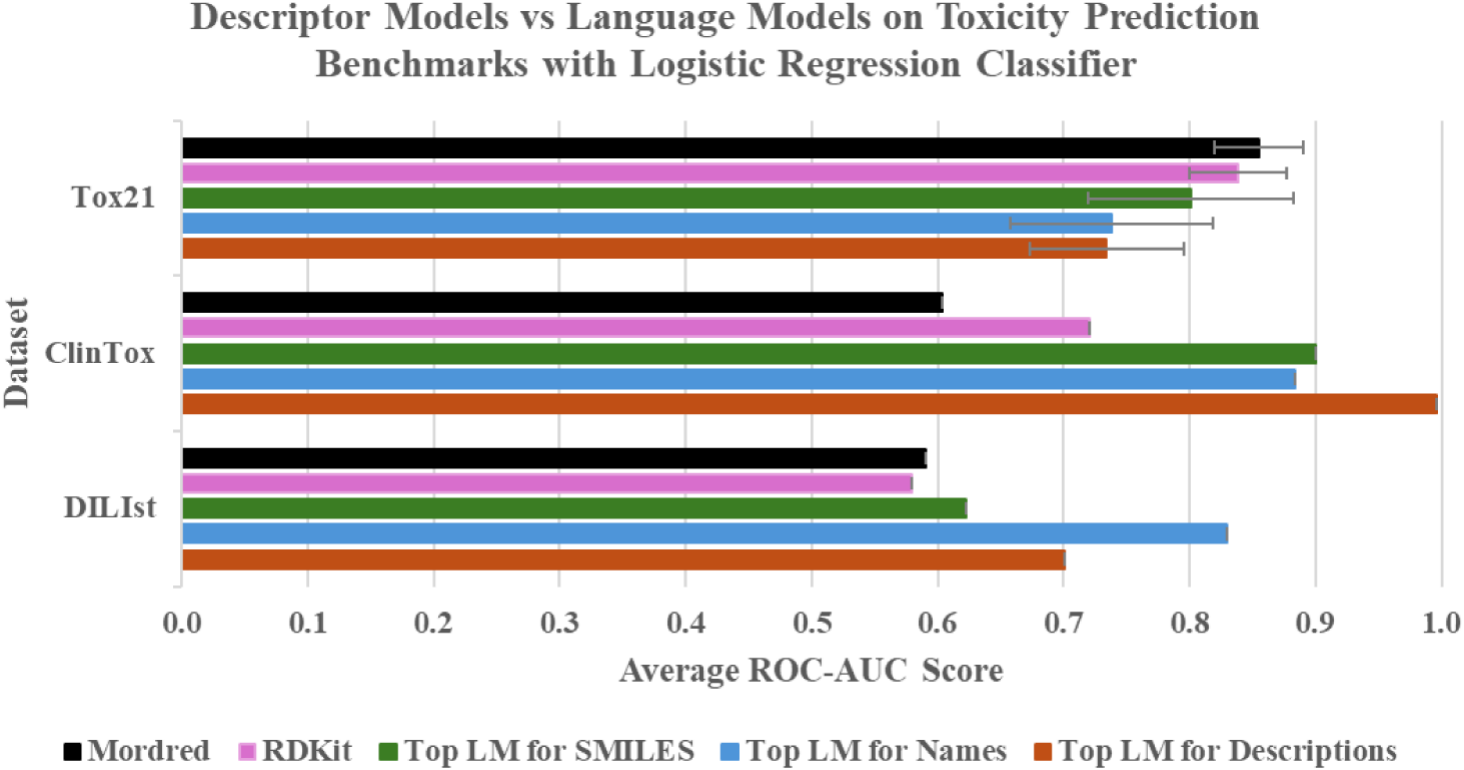
Bar chart of ROC-AUC scores on Tox21, ClinTox, and DILIst benchmarks across data sources and embedding methods for logistic regression classifier. Tox21: Top LM for SMILES = MolBERT, Top LM for Names = GPT-3, Top LM for Descriptions = Llama-3.1-8B. ClinTox: Top LM for SMILES = BERT, Top LM for Names = Llama-3.1-8B, Top LM for Descriptions = GPT-3. DILIst: Top LM for SMILES = Llama-3.2-1B, Top LM for Names = GPT-3, Top LM for Descriptions = Llama-3.1-8B.

## Discussion

This study systematically compared molecular descriptor-based models and language model-generated embeddings for toxicity prediction. We emphasize that this work is a systematic, reproducible methodology that goes beyond single-model comparisons: ten language models were evaluated across three distinct textual representations (SMILES, chemical names, and simple descriptions) alongside two standard descriptor sets (Mordred, RDKit), applying two classifier types (logistic regression, random forest), and testing across three toxicity datasets (Tox21, ClinTox, DILIst) using consistent preprocessing and splits. In addition, we report robust, nonparametric paired tests with multiple-comparison correction and provide the data needed to reproduce the results. These design choices enable fair, cross-model comparisons and yield practical guidance on when and where language-model embeddings add value.

The logistic regression results indicate that molecular descriptors remain an effective approach, with Mordred achieving the highest average ROC-AUC score (0.855) for Tox21, for instance. These findings reinforce the reliability of traditional cheminformatics methods that explicitly encode molecular properties relevant to toxicity assessment. The structured and interpretable feature engineering explains why descriptor-based models continue to serve as strong baselines in predictive toxicology. However, with the recent advancements in NLP and deep learning, language model embeddings surpass molecular descriptor-based models in multiple toxicity prediction tasks, including those of ClinTox and DILIst. The ability of AI models such as Llama-3 and GPT-3 to generate informative embeddings suggests that NLP-based approaches capture aspects of molecular structure and toxicity that may compete with traditional descriptors. In particular, for the ClinTox dataset, the top-performing language models surpassed RDKit, which obtained an ROC-AUC score of 0.721. Specifically, Llama-3.1-8B and GPT-3 outperformed this descriptor-based model by 0.162 and 0.275 when utilizing chemical names and simple descriptions, respectively. This indicates that language models can effectively encode toxicological information, even more efficiently than do descriptor-based models. These embeddings likely leverage vast pretraining on biomedical and chemical corpora to internalize patterns related to pharmacology, metabolism, and toxicity, thereby enabling them to generalize well even when limited structured data are available. Importantly, the performance gains observed for textual inputs such as chemical names or clinical descriptions underscore the value of language models in applications where molecular structure is inaccessible or poorly defined.

These results highlight the potential of NLP-driven embeddings to capture toxicity-related patterns in cases where predefined molecular descriptors may fall short, particularly for endpoints influenced by complex molecular interactions. While descriptor-based models remain a strong and interpretable approach, language models offer complementary strengths by encoding nuanced contextual information often missed by traditional features. This complementarity supports the development of hybrid approaches—such as ensemble models, late fusion techniques, or neural architectures that integrate both structured descriptors and unstructured embeddings—to improve predictive accuracy and robustness. As AI models continue to evolve with access to multimodal biomedical knowledge and expanded training datasets, their ability to generalize to diverse and challenging toxicity endpoints will likely improve. Additionally, fine-tuning language models on toxicology-specific corpora presents a promising direction for enhancing their relevance and effectiveness in endpoint-specific prediction tasks. In this study, embeddings were used “as-is” without task fine-tuning, which likely underutilizes the adaptability of chemical language models, and thus, fine-tuning on labeled toxicology data represents an important direction for future work.

Recent studies investigating LLMs and multimodal fusion for molecular property prediction report findings that align with our observations. For example, chain-of-thought-enabled LLMs combined with multimodal molecular fusion were shown to improve predictive accuracy and interpretability,^28^ LLM-Prop demonstrated that chemistry-aware pretraining can enhance generalization for property prediction,^29^ and the M2-LLM work finds benefits from multiview representation learning for molecular tasks.^30^ Consistent with these reports, our ablation shows small but generally positive gains from concatenating structured descriptors with LLM embeddings and a relative advantage for chemically pretrained models on structure-rich inputs; however, because we used fixed pretrained embeddings rather than task fine-tuning, direct numeric comparisons to fine-tuning studies should be made cautiously.

We note one important dataset limitation for Tox21: to ensure minimally stable per-endpoint evaluation, we retained only endpoints that initially contained at least 20 positive and 20 negative examples, and when more examples were available, we randomly sampled exactly 20 positives and 20 negatives to create a balanced per-endpoint set (40 compounds per endpoint). This procedure produced 29 Tox21 endpoints for analysis and reduces variability in per-endpoint ROC-AUC estimates, but it also substantially reduces the total training data and may limit generalizability to the original imbalanced distributions. We therefore emphasize that future work should evaluate models on the full, imbalanced datasets using imbalance-aware training (e.g., class weighting, resampling strategies, or loss functions such as focal loss) and larger sample sizes to assess robustness under real-world class imbalance; additionally, assessing extrapolation performance (for example, training on small molecules and evaluating on larger ones) represents an important avenue for future study.

### Dataset Characteristics and Their Influence on Model Performance

The observed variations in model performance across the Tox21, ClinTox, and DILIst datasets can, in part, be attributed to the distinct characteristics of these datasets. Tox21 differs from ClinTox and DILIst in that it consists of multiple toxicity-related endpoints, each representing a specific biological target or pathway, rather than a single binary classification of toxic or nontoxic compounds. This inherently increases the complexity of the predictive task, requiring models to capture diverse, endpoint-specific chemical patterns. Language model embeddings, which rely on textual representations rather than structured, property-based features, may struggle in this context due to the sparsity of endpoint-specific contextual information available in general language or chemical name corpora. Moreover, the scientific literature is unevenly distributed across Tox21 endpoints—well-studied pathways such as nuclear receptor activation may be better captured by language models, while less characterized or narrowly defined endpoints likely suffer from limited linguistic and contextual representation in the training data, leading to reduced performance.

In contrast, ClinTox and DILIst present single-endpoint classification tasks, simplifying the learning objective to a binary distinction between toxic and nontoxic compounds. This narrower focus may favor language models, as embeddings can encode more global, generalized, toxicity-relevant patterns from chemical names or descriptions without the need to differentiate between multiple, mechanistically distinct toxicities. The relatively high performance of language models on these datasets suggests that text-based representations can effectively capture broad toxicological risk when the prediction target is unified. Additionally, ClinTox and DILIst may reflect domains with richer public biomedical discourse surrounding toxicity, especially for known clinically toxic compounds, further improving the informativeness of language-derived features.

These differences emphasize the importance of dataset structure and endpoint diversity when evaluating machine learning models in toxicology. They also highlight an important limitation of language models in their current form: while effective for broad toxicity classifications, they may require additional fine-tuning or augmented domain-specific corpora to match the precision of molecular descriptors in multiendpoint prediction settings like Tox21. Beyond these structural differences, the varying performance of language models across datasets may also reflect differential representation of these benchmarks in pretraining corpora.

### Data Leakage Considerations

A critical limitation of this study concerns potential data leakage when evaluating pretrained language models on established benchmark datasets. Given that models like GPT-3 and Llama were trained on extensive web corpora, there is a high possibility that chemical compounds and their toxicity profiles from well-established public datasets, including ClinTox and DILIst, were encountered during pretraining. The high performance observed in GPT-3’s ClinTox performance (AUC = 0.996) likely reflects memorization and knowledge synthesis from previously seen data rather than genuine predictive capability.

This limitation fundamentally changes the interpretation of our results. Rather than demonstrating superior predictive modeling, the strong performance of LLMs on ClinTox and DILIst suggests that these approaches are most appropriately viewed as knowledge synthesis tools for well-characterized compounds, where existing toxicological information can be leveraged. In contrast, the superior performance of molecular descriptors on the multiendpoint Tox21 dataset—where many endpoints represent specialized biological assays with limited representation in general web corpora—likely reflects genuine predictive capability. These findings indicate that LLMs should be used cautiously in toxicological applications, particularly for regulatory submissions requiring demonstration of true predictive power, and are most suitable for preliminary screening of compounds with established toxicological profiles in the literature.

## Conclusion

Overall, this study underscores the continued importance of molecular descriptor-based models in toxicity prediction while recognizing the emerging role of language models as supplementary tools. While molecular descriptors demonstrated robust performance, particularly with Mordred achieving the highest average ROC-AUC score (0.855) on Tox21, language models showed competitive or superior performance on specific datasets, notably ClinTox and DILIst. However, a critical limitation concerns potential data leakage in pretrained language models, where the high performance likely reflects knowledge synthesis from previously encountered data. These findings suggest that language models should be viewed primarily as knowledge synthesis tools, most appropriately applied to well-characterized compounds where existing toxicological information can be leveraged, while molecular descriptors remain essential for genuine predictive modeling of novel compounds.

Future work should focus on refining multimodal strategies that combine both approaches, ensuring robust and interpretable toxicity predictions applicable to regulatory science and pharmaceutical research while addressing data leakage limitations through the development of truly novel test datasets. By harnessing the complementary strengths of molecular descriptors and language model embeddings, we can aim to advance the field of computational toxicology and contribute to safer drug development practices. These findings also highlight the growing opportunity for cross-disciplinary innovation—bringing together cheminformatics, natural language processing, and biomedical AI—to build next-generation models that are both accurate and explainable. Such approaches will be critical not only for predictive performance but also for earning the trust of end users in regulatory and clinical settings.

## Supplementary Material



## Data Availability

Code and processed data supporting this study are openly available at https://github.com/magnus-gray/Embedding4Tox.

## Notes

Additional figures and tables, including those pertaining to the random forest classifier (Figure S1), MAZ analysis (Table S1), and ablation study (Tables S2 and S3) (PDF)

## Abbreviations

AI: artificial intelligence
DILI: drug-induced liver injury
NLP: natural language processing
ROC-AUC: receiver operating characteristic – area under the curve
SMILES: Simplified Molecular Input Line Entry System

## References

[1] Fang H., Harris S. C., Liu Z., Zhou G., Zhang G., Xu J. (2016). FDA drug
labeling: rich resources to facilitate precision
medicine, drug safety, and regulatory science. Drug Discovery Today.

[2] Weininger D. (1988). SMILES, a
chemical language and information system. 1. Introduction to methodology
and encoding rules. J. Chem. Inf. Comput. Sci..

[3] Thomas R. S., Paules R. S., Simeonov A., Fitzpatrick S. C., Crofton K. M., Casey W. M. (2018). The
US Federal Tox21
Program: A strategic and operational plan for continued leadership. Altex.

[4] Wu Z., Ramsundar B., Feinberg E. N., Gomes J., Geniesse C., Pappu A. S. (2018). MoleculeNet: a benchmark for molecular machine
learning. Chem. Sci..

[5] Thakkar S., Li T., Liu Z., Wu L., Roberts R., Tong W. (2020). Drug-induced
liver injury severity and toxicity (DILIst): binary classification
of 1279 drugs by human hepatotoxicity. Drug
Discovery Today.

[6] Moriwaki H., Tian Y.-S., Kawashita N., Takagi T. (2018). Mordred: a molecular
descriptor calculator. J. Cheminf..

[7] Landrum G. (2013). RDKit: A software
suite for cheminformatics, computational chemistry, and predictive
modeling. Greg Landrum..

[8] Degtyarenko K., De Matos P., Ennis M., Hastings J., Zbinden M., McNaught A., Alcantara R., Darsow M., Guedj M., Ashburner M. (2007). ChEBI: a database
and ontology for chemical entities
of biological interest. Nucleic Acids Res..

[9] Edwards, C. ; Zhai, C. ; Ji, H. Text2mol: Cross-modal molecule retrieval with natural language queries. In Proceedings of the 2021 Conference on Empirical Methods in Natural Language Processing; ACL Anthology, 2021.

[10] Brown T., Mann B., Ryder N., Subbiah M., Kaplan J. D., Dhariwal P., Neelakantan A., Shyam P., Sastry G., Askell A. (2020). Language
models are few-shot learners. Adv. Neural Inf.
Process. Syst..

[11] Grattafiori, A. ; Dubey, A. ; Jauhri, A. ; Pandey, A. ; Kadian, A. ; Al-Dahle, A. ; Letman, A. ; Mathur, A. ; Schelten, A. ; Vaughan, A. The llama 3 herd of models arXiv 2024 10.48550/arXiv.2407.21783

[12] Chithrananda, S. ; Grand, G. ; Ramsundar, B. ChemBERTa: large-scale self-supervised pretraining for molecular property prediction arXiv 2020 10.48550/arXiv.2010.09885

[13] Fabian, B. ; Edlich, T. ; Gaspar, H. ; Segler, M. ; Meyers, J. ; Fiscato, M. Molecular representation learning with language models and domain-relevant auxiliary tasks arXiv 2020 10.48550/arXiv.2011.13230

[14] Ross J., Belgodere B., Chenthamarakshan V., Padhi I., Mroueh Y., Das P. (2022). Large-scale chemical language representations capture molecular structure
and properties. Nat. Mach. Intell..

[15] Devlin, J. ; Chang, M.-W. ; Lee, K. ; Toutanova, K. Bert: Pre-training of deep bidirectional transformers for language understanding arXiv 2019

[16] Wu L., Huang R., Tetko I. V., Xia Z., Xu J., Tong W. (2021). Trade-off predictivity and explainability
for machine-learning powered
predictive toxicology: an in-depth investigation with Tox21 data sets. Chem. Res. Toxicol..

[17] Wang Y., Xiao J., Suzek T. O., Zhang J., Wang J., Bryant S. H. (2009). PubChem: a public
information system for analyzing
bioactivities of small molecules. Nucleic Acids
Res..

[18] Devlin, J. ; Chang, M.-W. ; Lee, K. ; Toutanova, K. google-bert/bert-base-uncased; Hugging Face. https://huggingface.co/google-bert/bert-base-uncased.

[19] Chithrananda, S. ; Grand, G. ; Ramsundar, B. Seyonec /ChemBERTa-zinc-base-v1; Hugging Face; 2020. https://huggingface.co/seyonec/ChemBERTa-zinc-base-v1.

[20] ValizadehAslani, T. ; Shi, Y. ; Ren, P. ; Wang, J. ; Zhang, Y. ; Hu, M. , Lianglab/PharmBERT-uncased; Hugging Face; 2023. https://huggingface.co/Lianglab/PharmBERT-uncased.

[21] Wu, L. ; Gray, M. ; Dang, O. ; Xu, J. ; Fang, H. ; Tong, W. seldas/rxbert-v1; Hugging Face; 2023. https://huggingface.co/seldas/rxbert-v1. 10.1177/15353702231220669 PMC10798181 38166420

[22] Fabian, B. ; Edlich, T. ; Gaspar, H. ; Segler, M. ; Meyers, J. ; Fiscato, M. , BenevolentAI/MolBERT; GitHub; 2020. https://github.com/BenevolentAI/MolBERT.

[23] Ross, J. ; Belgodere, B. ; Chenthamarakshan, V. ; Padhi, I. ; Mroueh, Y. ; Das, P. ibm research/MoLFormer-XL-both--10pct; Hugging Face; 2022. https://huggingface.co/ibm-research/MoLFormer-XL-both-10pct.

[24] Grattafiori, A. ; Dubey, A. ; Jauhri, A. ; Pandey, A. ; Kadian, A. ; Al-Dahle, A. , meta llama/Llama-3.1–8B; Hugging Face; 2024. https://huggingface.co/meta-llama/Llama-3.1-8B.

[25] Grattafiori, A. ; Dubey, A. ; Jauhri, A. ; Pandey, A. ; Kadian, A. ; Al-Dahle, A. , meta -llama/Llama-3.2–1B, Hugging Face; 2024. https://huggingface.co/meta-llama/Llama-3.2-1B.

[26] Grattafiori, A. ; Dubey, A. ; Jauhri, A. ; Pandey, A. ; Kadian, A. ; Al-Dahle, A. , meta -llama/Llama-3.3–70B-Instruct; Hugging Face; 2024. https://huggingface.co/meta-llama/Llama-3.3-70B-Instruct.

[27] OpenAI New embedding models and API updates: OpenAI; 2024. https://openai.com/index/new-embedding-models-and-api-updates/.

[28] Jin C., Guo S., Zhou S., Guan J. (2025). Effective and Explainable Molecular
Property Prediction by Chain-of-Thought Enabled Large Language Models
and Multi-Modal Molecular Information Fusion. J. Chem. Inf. Model..

[29] Rubungo, A. N. ; Arnold, C. ; Rand, B. P. ; Dieng, A. B. LLM-Prop: predicting the properties of crystalline materials using large language models. arXiv. 2023.

[30] Ju, J. ; Zheng, Y. ; Koh, H. Y. ; Wang, C. ; Pan, S. M^2 LLM: Multi-view Molecular Representation Learning with Large Language Models. arXiv. 2025.

