# Supplementary material for: Comparative Study of Molecular Descriptors and AI-Based Embeddings for Toxicity Prediction
Source: Chem Res Toxicol. 2025 Nov 14;38(12):2061–70. doi: 10.1021/acs.chemrestox.5c00289 (PMC12709611; doi:10.1021/acs.chemrestox.5c00289)
Supplement: Supplementary file 1 [file tx5c00289_si_001.pdf]

Supplementary Materials for:

Comparative Study of Molecular Descriptors and AI-Based Embeddings for Toxicity Prediction

Magnus Gray[1], *Leihong Wu*[1*]

[1]Division of Bioinformatics and Biostatistics, National Center for Toxicological Research, U.S. FDA, 3900 NCTR Rd, Jefferson, AR, USA, 72079

Figure S1: Cross-Benchmark Random Forest Results

Figure S1 mirrors **Figure 5** from the main text, reporting the results for the random-forest classifier. It summarizes average ROC–AUC across Tox21, ClinTox, and DILIst for descriptor features and the top-performing language model per input modality.

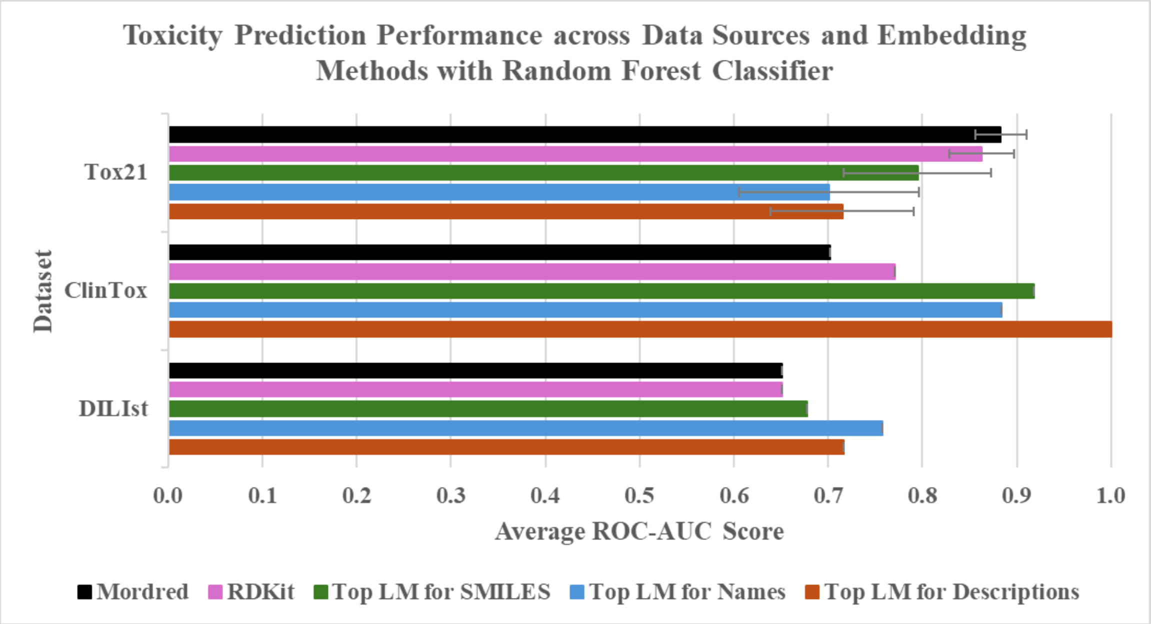

Figure S1: Bar Chart of ROC-AUC Scores on Tox21, ClinTox, and DILIst Benchmarks across Data Sources and Embedding Methods, for Random Forest Classifier. Tox21: Top LM for SMILES = MolBERT, Top LM for Names = GPT-3, Top LM for Descriptions = Llama-3.1-8B. ClinTox: Top LM for SMILES = BERT, Top LM for Names = Llama-3.1-8B, Top LM for Descriptions = GPT-3. DILIst: Top LM for SMILES = MolBERT, Top LM for Names = Llama-3.3-70B-Instruct, Top LM for Descriptions = Llama-3.2-1B.

Table S1: Tox21 MAZ Results

Table S1 contains the complete MAZ values used in the Tox21 analysis, using the logistic regression classifier. Lower MAZ indicates closer alignment of LM endpoint-level ROC–AUCs to descriptor models (Mordred, RDKit). The MAZ definition and formulas appears in Methods of the main text.

Table S1: Mean Absolute Z-score (MAZ) of Language Models Compared to Molecular Descriptor-Based Models, for Logistic Regression Classifier, Across Textual Data Types.

Language Model	SMILES Strings		Chemical Names		Simple Descriptions	
	Mordred	RDKit	Mordred	RDKit	Mordred	RDKit
BERT	3.06	2.55	7.31	6.20	6.61	5.57
ChemBERTa	3.94	3.22	--	--	--	--
PharmBERT	3.13	2.50	6.90	5.84	6.00	5.01
RxBERT	2.82	2.31	6.08	5.09	5.27	4.35
MolBERT	**2.18**	**2.00**	--	--	--	--
MoLFormer	3.05	2.56	--	--	--	--
Llama-3.2-1B	3.68	2.96	6.84	5.78	3.69	3.03
Llama-3.1-8B	3.10	2.61	5.77	4.81	**3.53**	**2.85**
Llama-3.3-70B-Instruct	2.89	2.40	5.91	5.00	3.71	3.15
GPT-3	3.64	3.11	**3.76**	**3.00**	3.74	3.04

Table S2: Ablation Analysis of Fusion Features

Table S2 reports the aggregate ablation results on DILIst, using the logistic regression classifier. The table shows the mean (SD) ROC–AUC scores over five seeds, including runs that employ simple descriptor and embedding fusion ("+ RDKit").

Table S2: Ablation Results on DILIst: Mean (SD) ROC-AUC Scores for Logistic Regression Classifier Across Five Seeds, Examining Fusion Features; The Embedding Types "SMILES + RDKit", "Names + RDKit", and "Descriptions + RDKit" Indicate Concatenation of the Language-Model Embedding with RDKit Descriptors.

Model	Embedding Type	ROC-AUC Scores [Mean (SD)]
**RDKit**	*RDKit*	0.618 (0.037)
**Mordred**	*Mordred*	0.566 (0.031)
**BERT**	*SMILES*	0.557 (0.046)
	Names	0.527 (0.025)
	Descriptions	0.588 (0.028)
	SMILES + RDKit	0.601 (0.017)
	Names + RDKit	0.571 (0.020)
	Descriptions + RDKit	0.602 (0.030)
**ChemBERTa**	*SMILES*	0.562 (0.055)
	Names	0.575 (0.038)
	Descriptions	0.558 (0.026)
	SMILES + RDKit	0.566 (0.051)
	Names + RDKit	0.610 (0.011)
	Descriptions + RDKit	0.577 (0.025)
**PharmBERT**	*SMILES*	0.570 (0.040)
	Names	0.595 (0.022)
	Descriptions	0.594 (0.027)
	SMILES + RDKit	0.582 (0.018)
	Names + RDKit	0.623 (0.011)
	Descriptions + RDKit	0.601 (0.013)
**RxBERT**	*SMILES*	0.556 (0.038)
	Names	0.680 (0.043)
	Descriptions	0.627 (0.025)
	SMILES + RDKit	0.589 (0.006)
	Names + RDKit	0.675 (0.029)
	Descriptions + RDKit	0.616 (0.014)
**MolBERT**	*SMILES*	0.556 (0.051)
	Names	0.500 (0.000)
	Descriptions	0.501 (0.002)
	SMILES + RDKit	0.588 (0.029)

	Names + RDKit	0.577 (0.026)
	Descriptions + RDKit	0.579 (0.028)
**MoLFormer**	*SMILES*	0.619 (0.030)
	Names	0.590 (0.015)
	Descriptions	0.553 (0.041)
	SMILES + RDKit	0.593 (0.010)
	Names + RDKit	0.585 (0.016)
	Descriptions + RDKit	0.581 (0.024)
**Llama-3.2-1B**	*SMILES*	0.618 (0.021)
	Names	0.740 (0.035)
	Descriptions	0.641 (0.021)
	SMILES + RDKit	0.600 (0.023)
	Names + RDKit	0.724 (0.034)
	Descriptions + RDKit	0.644 (0.016)
**Llama-3.1-8B**	*SMILES*	0.605 (0.020)
	Names	0.790 (0.034)
	Descriptions	0.696 (0.021)
	SMILES + RDKit	0.606 (0.022)
	Names + RDKit	0.778 (0.041)
	Descriptions + RDKit	0.669 (0.015)
**Llama-3.3-70B-Instruct**	*SMILES*	0.526 (0.032)
	Names	0.774 (0.035)
	Descriptions	0.633 (0.023)
	SMILES + RDKit	0.569 (0.032)
	Names + RDKit	0.777 (0.036)
	Descriptions + RDKit	0.642 (0.019)
**GPT-3**	*SMILES*	0.539 (0.038)
	Names	0.813 (0.041)
	Descriptions	0.682 (0.013)
	SMILES + RDKit	0.558 (0.045)
	Names + RDKit	0.788 (0.043)
	Descriptions + RDKit	0.670 (0.021)

Table S3: Compound-Level Case Study of DILIst Results

Table S3 highlights representative compound-level cases where descriptor models failed but the language models for a given input type typically succeeded for the DILIst dataset. This table illustrates the hypothesized lexical/class-level cues leveraged by the language models that led to their success.

Table S3: Sample of Cases where Descriptor Models Fail but Language Models Succeed on the DILIst dataset.

Compound Name	SMILES	Description Snippet	LM Input Type	Hypothesis for Prediction Difference
Glucosamine	C(C1C(C(C(C(O1)O)N)O)O)O	2-amino-2-deoxy-D-glucopyranose … has a role as an Escherichia coli metabolite, a mouse metabolite and a geroprotector.	Names	The compound name "glucosamine" provides a direct lexical cue linked to known bioactivity, which LMs can exploit but descriptors cannot.
Melphalan	C1=CC(=CC=C1CC(C(=O)O)N)N(CCCl)CCCl	Melphalan … has a role as an antineoplastic agent, a carcinogenic agent, an alkylating agent, an immunosuppressive agent and a drug allergen.	Descriptions	The text description highlights drug class terms (e.g., "alkylating agent") that LMs associate with toxicity, whereas descriptors miss this lexical signal.
Thiabendazole	C1=CC=C2C(=C1)NC(=N2)C3=CSC=N3	Thiabendazole is a member of the class of benzimidazoles … [and] has a role as an antifungal agrochemical and an antinematodal drug.	Names	The name signals class membership ("benzimidazole"), providing a clear toxicity-linked cue absent from numeric descriptors.
Arbekacin	C1CC(C(OC1CN)OC2C(CC(C(C2O)OC3C(C(C(C(O3)CO)O)N)O)NC(=O)C(CCN)O)N)N	Arbekacin is a kanamycin that … has a role as an antimicrobial agent, a protein synthesis inhibitor, an antibacterial agent and an antibacterial drug.	Names	The name signals class membership ("aminoglycoside"), which LMs leverage to infer toxicity associations unavailable to descriptors.
